# Central nervous system sulfatide deficiency as a causal factor for bladder disorder in Alzheimer's disease

**DOI:** 10.1002/ctm2.1332

**Published:** 2023-07-21

**Authors:** Sijia He, Shulan Qiu, Meixia Pan, Juan P. Palavicini, Hu Wang, Xin Li, Anindita Bhattacharjee, Savannah Barannikov, Kevin F. Bieniek, Jeffrey L. Dupree, Xianlin Han

**Affiliations:** ^1^ Barshop Institute for Longevity and Aging Studies University of Texas Health San Antonio San Antonio Texas USA; ^2^ Division of Diabetes Department of Medicine University of Texas Health San Antonio San Antonio Texas USA; ^3^ Department of Pathology Glenn Biggs Institute for Alzheimer's and Neurodegenerative Diseases University of Texas Health San Antonio San Antonio Texas USA; ^4^ Department of Anatomy and Neurobiology Virginia Commonwealth University Richmond Virginia USA; ^5^ Research Division McGuire Veterans Affairs Medical Center Richmond Virginia USA

**Keywords:** Alzheimer's disease, bladder, lipidome, spinal cord, sulfatide

## Abstract

**Background:**

Despite being a brain disorder, Alzheimer's disease (AD) is often accompanied by peripheral organ dysregulations (e.g., loss of bladder control in late‐stage AD), which highly rely on spinal cord coordination. However, the causal factor(s) for peripheral organ dysregulation in AD remain elusive.

**Methods:**

The central nervous system (CNS) is enriched in lipids. We applied quantitative shotgun lipidomics to determine lipid profiles of human AD spinal cord tissues. Additionally, a CNS sulfatide (ST)‐deficient mouse model was used to study the lipidome, transcriptome and peripheral organ phenotypes of ST loss.

**Results:**

We observed marked myelin lipid reduction in the spinal cord of AD subjects versus cognitively normal individuals. Among which, levels of ST, a myelin‐enriched lipid class, were strongly and negatively associated with the severity of AD. A CNS myelin‐specific ST‐deficient mouse model was used to further identify the causes and consequences of spinal cord lipidome changes. Interestingly, ST deficiency led to spinal cord lipidome and transcriptome profiles highly resembling those observed in AD, characterized by decline of multiple myelin‐enriched lipid classes and enhanced inflammatory responses, respectively. These changes significantly disrupted spinal cord function and led to substantial enlargement of urinary bladder in ST‐deficient mice.

**Conclusions:**

Our study identified CNS ST deficiency as a causal factor for AD‐like lipid dysregulation, inflammation response and ultimately the development of bladder disorders. Targeting to maintain ST levels may serve as a promising strategy for the prevention and treatment of AD‐related peripheral disorders.

## Graphical abstract

Shotgun lipidomics identified a unique lipid signature in Alzheimer's disease (AD) versus normal spinal cord characterized by a significant decrease in myelin lipids. Depletion of central nervous system (CNS) sulfatide (ST) in mice led to spinal cord lipidomes and transcriptomes highly resemble those of human AD. Prolonged CNS ST loss causes neurogenic bladder. Altered lipid metabolism contributes to ST loss‐induced myelin lipidome disruption.

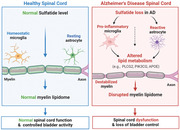

## 1 BACKGROUND

Alzheimer's disease (AD) is the most common form of dementia that manifests as a decline in memory and cognition resulting in impaired activities of daily living. Scientific research on AD pathophysiology has been mainly concentrated on the brain, however, it is widely recognized that AD‐related peripheral secondary diseases, such as pneumonia, injury and sepsis due to urinary tract infections, play a major role in mortality of AD patients,^1^, ^2^, ^3^ underscoring the urgent need to understand the causes and molecular mechanisms of AD‐associated peripheral deficits.

Lower urinary tract dysfunction (LUTD) such as urinary incontinence is prevalent in middle‐ to late‐stage AD.^4^, ^5^, ^6^ Although cognitive impairment has been proposed to be associated with abnormal urinary incidents,^7^ the underlying mechanistic connection between AD development and the associated bladder disorders is unknown. Recent studies have brought to attention the involvement of spinal cord in AD pathogenesis.^8^ As an extension of the brain, the spinal cord is a pivotal part of the central nervous system (CNS) that functions as a bridge between the brain and various parts of the body.^9^ Disruption of spinal cord function in AD has been described by several studies.^8^ Clinical symptoms such as fine motor impairment appear in early AD prior to cognitive dysfunction, suggesting spinal cord‐regulated sensorimotor network rewiring during initial disease stages.^10^ Spinal cord atrophy is a proposed in vivo imaging marker of AD dementia.^8^ Amyloid beta pathology and phosphorylated tau tangles are present in the spinal cord of post‐mortem AD patients^11^, ^12^ and AD animal models.^13^, ^14^ In addition, spinal cord injury (SCI) increases the risk of AD development.^15^ These studies strongly suggest a connection among spinal cord dysfunction, AD‐related peripheral dysregulation and AD progression. However, the molecular mechanisms driving this process remain elusive.

The CNS is highly enriched in lipids that are critical for membrane fluidity control, electrical signal transmissions and second messenger generation.^16^ Abnormal lipid metabolism is commonly observed in aging and pathological conditions, and it is known to contribute to the onset of neurodegenerative disorders.^17^, ^18^ Using a lipidomics approach, we previously published that sulfatide (ST), a class of myelin glycosphingolipids, is substantially lost in post‐mortem human AD brains.^19^, ^20^ Using a mouse model of adult‐onset myelin‐specific ST deficiency, we recently demonstrated that ST loss is sufficient to cause neuroinflammation,^18^, ^21^ myelin and axonal disorganization,^22^ brain ventricular enlargement,^23^ and cognitive impairment resembling AD.^18^

To broaden our understanding of spinal cord involvement in AD progression, herein, we investigated lipidome alterations in AD spinal cord using quantitative shotgun lipidomics. Further, we explored spinal cord–peripheral consequences and related molecular mechanisms of adult‐onset ST deficiency using a mouse model of myelinating cell‐specific depletion (driven by Plp1‐CreERT) of cerebroside (CBS) sulfotransferase (*CST*, a.k.a. *Gal3st1*) gene, which codes for the final‐step enzyme that catalyses ST biosynthesis. Our results demonstrated that ST decline triggers a marked loss of myelin lipids in the spinal cord, which disrupt its proper coordination of peripheral organs (such as urinary bladder). Our study and mouse model provide a mechanistic tool for understanding the link between the CNS disease progression and peripheral disorders (such as late‐stage bladder/bowel disorder) in human AD.

## 2 METHODS

### 2.1 Study design and approval

Human spinal cord samples were provided by the NIH NeuroBioBank (nine cases) and the Glenn Biggs BrainBank of UT Health SA (nine cases). Written consents were obtained for the use of human spinal cord samples for research. Frozen human spinal cord samples were kept in a −80°C freezer for storage upon receipt before 10–30 mg tissue was sectioned for use to measure gene expression, and protein and lipid levels, respectively.

For animal studies, experiments were performed in accordance with the guidelines approved by the Institutional Animal Care and Use Committee (IACUC) of University of Texas Health San Antonio (UT Health SA). We established the number of animals for different experimental conditions based on the variance observed in preliminary experiments. No data were excluded, and outliers are presented and included in the analyses. Animals and other experimental units were assigned randomly to the experimental groups. The sample size for in vivo experiment was based on power calculations and is indicated in the figure legends.

### 2.2 Animal study

Mice were housed under 12/12 h light/dark cycles with free access to food and water. Mice were fed Teklad laboratory diet (ENVIGO, Cat. #7012). *CST* ^fl/fl^ mouse was generated as described in our previous study^18^ by flanking exon 3 and 4 of the *Gal3st1* gene with LoxP sequences. To generate oligodendrocyte‐specific *Gal3st1* knockout mouse, *CST* ^fl/fl^ mice were crossed with *Plp1* ^CreERT/+^ mice (Stock No: 005975, the Jackson Laboratory, Bar Harbor, ME, USA). *CST* ^fl/fl^; *Plp1* ^+/+^ mice (Loxp) and *CST* ^fl/fl^; *Plp1* ^CreERT/+^ mice (CKO) were injected with tamoxifen (40 mg/kg bodyweight) at 3–4 month old to induce knockout of *CST* gene post developmental stage. Data represent results from both sexes unless specified in figure legends. For the body composition study, quantitative magnetic resonance imaging (qMRI) was performed using EchoMRI by the Healthspan and Functional Assessment Core of UT Health SA. For the urine collection study, mice were housed individually for 24 h with free access to food and water, urine and faecal samples were routed into separate collection cups. Urine samples were centrifuged at 10 000 × *g* for 1 min and the supernatant was stored in −80°C freezer until use. Frailty was evaluated according to the method described previously^24^ using a performance‐based eight‐item frailty index system.

### 2.3 Nerve conduction studies

Nerve conduction velocity (NCV) was conducted based on the method previously described^25^ using the Nicolet Viking Quest portable EMG apparatus (CareFusion, San Diego, CA). One hertz low frequency filters and 10 kHz high frequency filters were used. Mice were anesthetized with stable flow isoflurane while body temperature was maintained at 34°C using a heating lamp. Impulse stimulations (0.02 ms) were delivered supramaximal using stainless steel subdermal needle electrodes. For tail nerve evaluation, stimulating electrodes and recording electrodes were inserted in the tail at 3‐cm apart. The tail motor action potential latency and amplitude were measured by stimulating proximally and recording distally, while the tail sensory NCV was calculated by stimulating distally and recording proximally. To measure the sciatic NCV, the ankle–foot latency was first recorded by stimulating at the ankle and recording dorsally at all five digits, the latency and distance between electrodes were recorded before moving stimulating electrodes to the sciatic notch, where the nerve was stimulated again. The resulting latency was subtracted by the initial ankle–foot latency, the velocity was calculated using the difference of latency divided by the distance between notch and ankle.

### 2.4 Spinal cord sample preparation

For human spinal cord samples, lumbar spinal cord was dissected into white and grey matter for lipidomics, while sacral spinal cord samples were analysed as a mixture of white and grey matter. Frozen tissue was cryofractured in liquid nitrogen and split for protein, mRNA and lipid analyses. For mouse spinal cord, all animal were anesthetized using isoflurane and perfused with saline. Whole spines were isolated and incubated in 4% paraformaldehyde (PFA) with rotation for overnight fixation at 4°C. Whole spinal cords were then carefully isolated from vertebras, washed with PBS and placed sequentially through 10%, 20% and 30% sucrose solution, and embedded into frozen blocks. For mRNA, protein and lipid analysis, whole spinal cord was isolated upon sacrifice, flash frozen in liquid nitrogen and stored in a −80°C freezer until use.

### 2.5 Lipid extraction and lipidomics measurements

Lipid analysis was performed using multi‐dimensional mass spectrometry‐based shotgun lipidomics as previously described.^26^ Briefly, frozen samples were weighed and homogenized in diluted PBS. Protein concentration was determined using bicinchoninic acid (BCA) assay. An equal amount of homogenate from individual samples based on protein content was used for lipid extraction using a modified procedure of Bligh and Dyer extraction in the presence of internal standards. The levels of lipid molecular species were assessed using a triple‐quadrupole mass spectrometer (Thermo Fisher Scientific, Waltham, MA, USA) equipped with a Nanomate device (Advion, Ithaca, NY, USA) and Xcalibur system as previously described.^27^ Subsequent data processing was conducted using a custom‐programmed Microsoft Excel macro as previously described.^28^

### 2.6 mRNA extraction and gene expression analysis

RNA from frozen, powdered spinal cord samples were extracted using the simplyRNA Tissue kit (Maxwell RSC, Cat. # AS1340) with initial homogenization by TissueLyser LT (QIAGEN) per manufacturer's instructions. Following extraction, RNA was eluted in a total of 50 μL of nuclease‐free water. Sample concentration was determined using the Qubit RNA BR Assay Kit (Thermo Fisher Scientific, Cat. # Q32852), RNA integrity (RIN) was determined using the 4150 TapeStation system (Agilent) via RNA ScreenTapes. Gene expression was assessed using NanoString nCounter Technology using mouse or human Neuroinflammation panels and nCounter SPRINT Profiler per manufacturer's instructions (NanoString Technologies). Initial data analysis including background thresholding at mean value of negative controls, positive control normalization and codeSet content normalization was performed using nSolver Analysis Software 4.0. Further analysis was performed via nSolver Advanced Analysis Software 2.0, where normalized data were used to complete fold change analysis between groups. For qPCR analysis, gene expression levels were detected using SYBR Green (Applied Biosystems, Cat. #A25742) method using primer sequences listed in Table S1. The relative gene expression was normalized to endogenous housekeeping gene GAPDH levels using the ΔΔCT method, and data were presented as fold changes over respective controls.

### 2.7 Immunoblotting analysis

Frozen samples were cryofractured in liquid nitrogen, weighed and homogenized in N‐PER Neuronal Protein Extraction Reagent (Thermo Fisher Scientific, Cat. # 87792) containing protease and phosphatase inhibitors (Thermo Fisher Scientific, Cat. # 78442). After 10‐min incubation on ice, samples were centrifuged at 12 000 × *g* for 15 min at 4°C followed by protein quantification using BCA method. Thirty μg protein/sample was loaded into NuPAGE 4%−12% Bis–Tris gels and resolved using the NuPAGE electrophoresis system (Life technologies). Samples were transferred onto PVDF membrane, blocked in 1% bovine serum albumin at room temperature for 1 h and incubated with primary antibodies at 4°C overnight. The blots were then incubated with horseradish peroxidase (HRP)‐conjugated secondary antibody and developed using the chemiluminescence (ECL) method (Thermo Fisher Scientific, Cat. # 32106). Table S2 contains a list of antibodies used.

### 2.8 Immunofluorescence staining

Serial 10‐μm cross sections of the frozen spinal cord were collected and rinsed in PBS. After 1‐h blocking with 10% goat serum (Sigma, Cat. # G9023) at room temperature, sections were incubated with primary antibody diluted in PBS‐T (PBS containing 0.05 Triton X‐100) with 5% goat serum at 4°C overnight in a humidified chamber. Slides were washed three times in PBST, incubated with fluorescence‐labelled second antibody for 1 h at room temperature, washed again before being mounted using ProLong^™^ Diamond Antifade Mountant with DAPI (Thermo Fisher Scientific, Cat. # P36971). Images were collected using a fluorescence microscope (KEYENCE, Cat. # BZ‐X800).

### 2.9 Statistics

All data were shown as mean ± standard error of the mean (SEM) unless specified. For animal experiments, age and sex‐matched mice were assigned randomly to different treatment groups to prevent potential bias. All biochemistry results were representative of at least three repeated experiments or as indicated. Statistical analysis was performed using GraphPad Prism 9 and Microsoft Excel. Principal component analysis (PCA) was performed using MetaboAnalyst 5.0. Unpaired two‐tailed *t*‐test was used for the comparison between two groups. One‐way ANOVA followed with Tukey's test was performed to compare multiple groups. *p* ≤ .05 was considered statistically significant.

## 3 RESULTS

### 3.1 Substantial decline of myelin lipids and increased neuroinflammation in spinal cord tissue of AD subjects compared to controls

**FIGURE 1:**
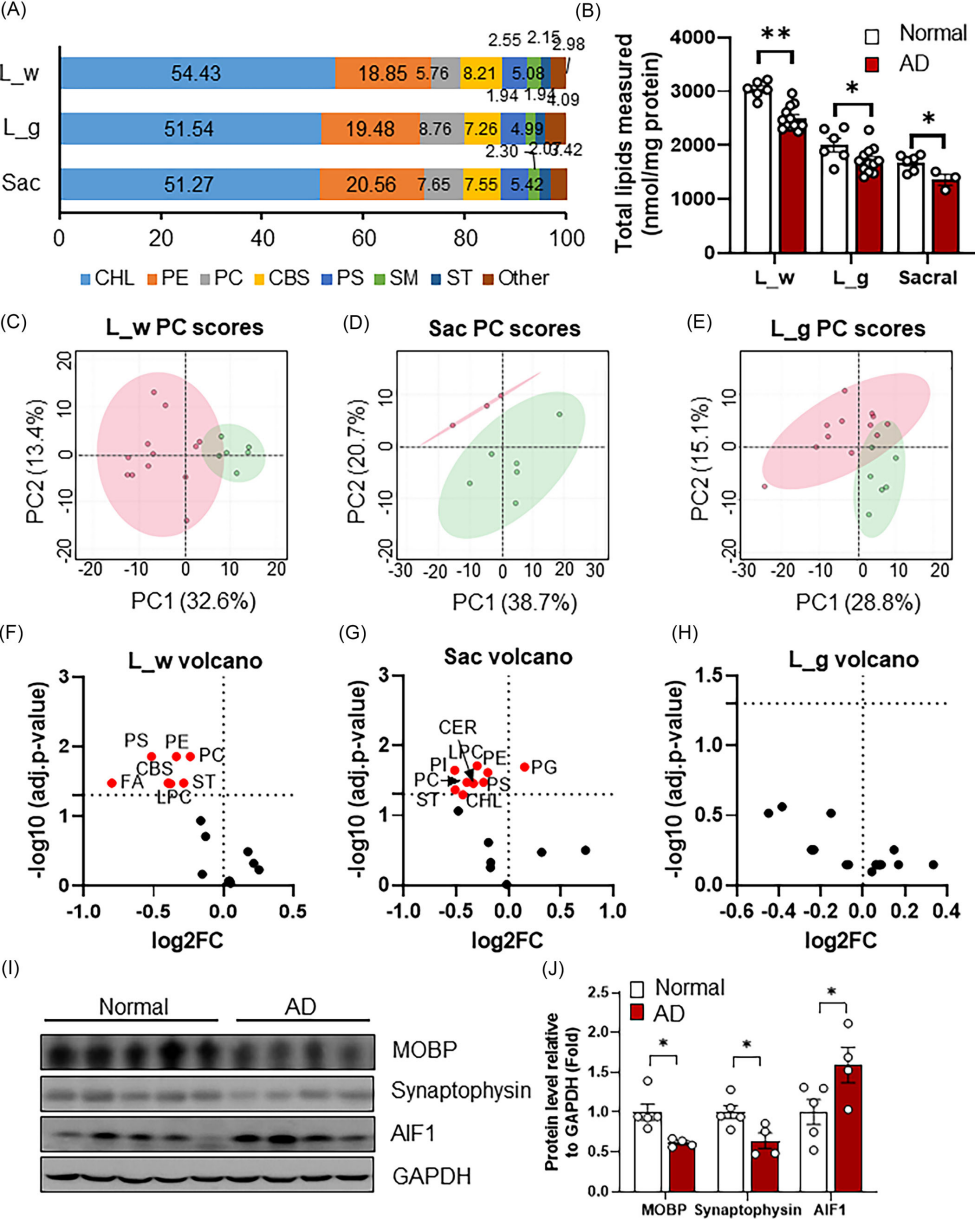
Disrupted lipid profiles and cell type‐specific protein markers in the spinal cord tissue samples of Alzheimer's disease (AD) subjects compared to normal individuals. (A) Percentage content of major lipid species from human spinal cord tissues measured by shotgun lipidomics (data were from control and AD group combined). L_g: lumbar grey matter; L_w, lumbar white matter; Sac, sacral. (B) Total lipid content of spinal cord tissues quantified in relative to protein levels. Principle component analysis (PCA) of lipidome for lumbar white matter (C), sacral spinal cord (D) and lumbar grey matter (E) according to Alzheimer's disease neuropathologic change (ADNC) pathology. Green colour: Normal (*n* = 6), pink colour: ADNC (*n* = 12 for L_w and L_g, *n* = 3 for Sac). Levels of each lipid group from samples with ADNC compared to normal status in lumbar white matter (F), sacral spinal cord (G) and lumbar grey matter (H), red coloured dot indicate lipid that reached significant change (adjusted *p* < .05). For lumbar cord, normal (*n* = 6), AD (*n* = 12); for sacral cord, normal (*n* = 6), AD (*n* = 3). (I) Western‐blot detection of protein levels of oligodendrocyte, microglia and neuron markers in human lumbar spinal cord samples (mixture of white and grey matter, *n* = 5 for normal group, *n* = 4 for ADNC group). (J) Quantification of (I) based on band intensity using image J software. Data represent mean ± SEM in (B) and (J). Unpaired two‐tailed *t*‐test, *p ≤ .05, **p ≤ .01. CBS, cerebroside; CER, ceramide; CHL, cholesterol; FA, fatty acyl chains in TAG; LPC, lyso‐PC; LPE, lyso‐PE; PC, phosphatidylcholine; PA, phosphatidic acid; PE, phosphatidylethanolamine; PG, phosphatidylglycerol; PI, phosphatidylinositol; PS, phosphatidylserine; SM, sphingomyelin; ST, sulfatide; TAG, triacylglycerol.

To determine the lipid profiles of spinal cord tissue and explore the potential impact of lipid alterations on AD, post‐mortem spinal cord samples from individuals with/without Alzheimer's disease neuropathologic change (ADNC) were subjected to shotgun lipidomics. The demographical and sample information are listed in Table 1. Lumbar spinal cord was separated into white (L_w) and grey (L_g) matter, sacral spinal cord (Sac) was measured as a mixture of white and grey matter. Lipidomics identified and quantified a total of 284 lipid molecular species scanned from thousands of molecular species, which were categorized into 18 lipid classes (Figure S1A). Among which, cholesterol (CHL) is the most abundant (51.27%–54.43%), followed by phosphatidylethanolamine (PE) (18.85%–20.56%), phosphatidylcholine (PC) (5.76%–8.76%), CBS (7.26%–8.21%), phosphatidylserine (PS) (4.99%–5.42%), sphingomyelin (SM) (1.94%–2.55%), ST (1.94%–2.15%) and others (2.98%–4.09%) (Figure 1A). While the composition of lipids remains largely unchanged in AD versus normal (Figure S1B), marked reduction of total lipid content was observed in AD groups (Figure 1B). Consistently, levels of both saturated and unsaturated fatty acid chains were declined (Figure S1C,D), and decreased unsaturation index in polyunsaturated fatty acids (Figure S1E) was present in AD L_w versus controls. PCA showed clear separation of normal and AD samples (Figure 1C–E). Further comparison detected significant reduction of multiple lipid classes including ST, PS, PE, PC, Lyso‐PC in both L_w and Sac samples of AD group (Figure 1F,G), but not in L_g samples (Figure 1H). These results identified a substantial loss of lipids in the spinal cord of AD subjects compared to cognitively normal individuals, which was mainly contributed by myelin‐enriched white matter.

**TABLE 1:** Demographic profile of human spinal cord sample cases.

Pathology	Control	ADNC	*p*‐value
Braak	Normal	I–VI	−
**Age (years)** Mean ± SD	60.16 ± 11.72	73.66 ± 14.23	.062752
**Sex**	Male = 3; Female = 3	Male = 5; Female = 7	−
**Race**	White	White	−
**PMI(h)** Mean ± SD	23.66 ± 2.16	14.62 ± 7.90	.0153
**Complications**	Cardiac arrhythmia; atherosclerosis heart disease; aortic aneurysm; pulmonary thromboembolus; CVD	CVD; CAA; ALS; dementia with Lewy bodies; glioblastoma	−

Abbreviations: ALS, amyotrophic lateral sclerosis; CAA, cerebral amyloid angiopathy; CVD, cardiovascular diseases; PMI, post‐mortem interval; ADNC, Alzheimer's disease neuropathologic change.

Increased gliosis and synaptic dysfunction are among major characteristics of AD brain,^29^, ^30^ yet few studies have explored such pathological changes in spinal cord. We detected significant loss of synaptophysin, a presynaptic vesicle marker, in AD versus control (Figure 1I,J). On the contrary, microglial marker allograft inflammatory factor 1 (AIF1, a.k.a. Iba1) was greatly induced in AD spinal cord, suggesting increased microglial activation. Myelin‐associated oligodendrocyte basic protein (MOBP), a CNS myelin‐specific protein, was largely decreased in AD versus controls (Figure 1I,J), indicating myelin sheath destabilization.

### 3.2 Marked ST deficiency in the spinal cord of AD subjects

**FIGURE 2:**
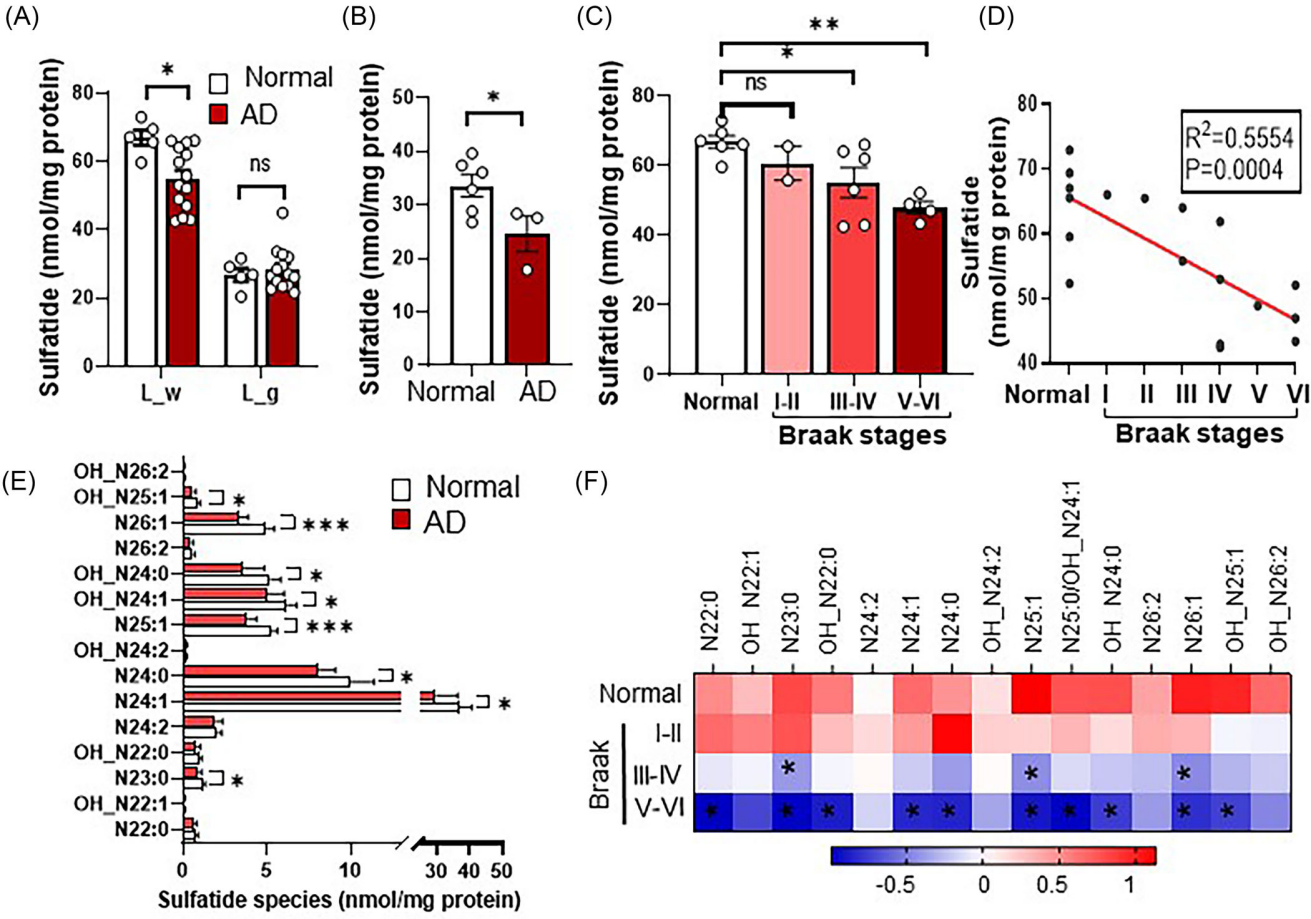
Marked reduction of sulfatide (ST) in human spinal cord tissue from Alzheimer's disease (AD) patients versus controls. (A) Total ST content in human lumbar spinal cord tissues measured by shotgun lipidomics (*n* = 6 for normal, *n* = 12 for AD). (B) Total ST content in human sacral spinal cord tissues measured by shotgun lipidomics (*n* = 6 for normal, *n* = 3 for AD). (C) ST levels in the white matter of lumbar spinal cord grouped according to Braak stages (*n* = 6, 2, 6 and 4 for normal, Braak I–II, III–IV and V–VI, respectively). (D) Association analysis of lumbar white matter ST levels with Braak stages (*n* = 18). (E) Levels of lumbar white matter ST species with comparison between normal (*n* = 6) and AD (Braak ≥ III, *n* = 10) samples. (F) Heatmap displays levels of each ST lipid species from samples with AD neuropathologic change of different Braak stages compared to normal status. Data represent mean ± SEM for (A), (B), (C) and (E). Unpaired two‐tailed *t*‐test for (A), (B) and (E). One‐way ANOVA with Dunnett correction for (C), *p ≤ .05, **p ≤ .01, ***p ≤ .001.

Despite being conceptualized as grey matter disease,^31^ AD has been associated with multiple aspects of white matter disorders.^32^ Interestingly, here we observed a more severe impact on white matter than grey matter at the lipidome level in AD spinal cord (Figure 1F,H). To further explore the correlation between lipid alterations and AD progression in spinal cord tissue, we focused our investigation on white matter enriched lipid classes and molecular species that play a role in AD. Several lipid species, including CBS and CHL (Figure S1F,G) were of potential interest. However, we focused our investigation on ST due to its substantial decline in very early clinical stages of AD brain tissue, preceding changes of other lipid species.^20^ Moreover, ST has well‐established interaction with multiple AD risk factors^33^ and plays a critical role in modulating cognitive function.^18^ Based on these reasons, we hypothesized that ST levels may be negatively correlated with AD severity in spinal cord tissues. As expected, lipidomics data show that ST content is highly enriched in white matter compared to grey matter and interestingly, was significantly decreased in L_w tissue samples of AD patients versus controls (Figure 2A). A similar result was seen in Sac tissue (Figure 2B). Moreover, the decline of ST levels in spinal cord was strongly correlated with the Braak neurofibrillary tangle stages of AD (Figure 2C,D), suggesting that ST loss in AD spinal cord is associated with an increased severity of AD tau pathology. Further, among multiple ST species detected, the most abundant species including N24:1, N24:0, N25:0, N25:1 and N26:1 were greatly declined in samples from more advanced Braak stages (Figure 2E,F).

### 3.3 Adult‐onset ST loss leads to the loss of myelin lipids and promotes neuroinflammation in the mouse spinal cord resembling AD

**FIGURE 3:**
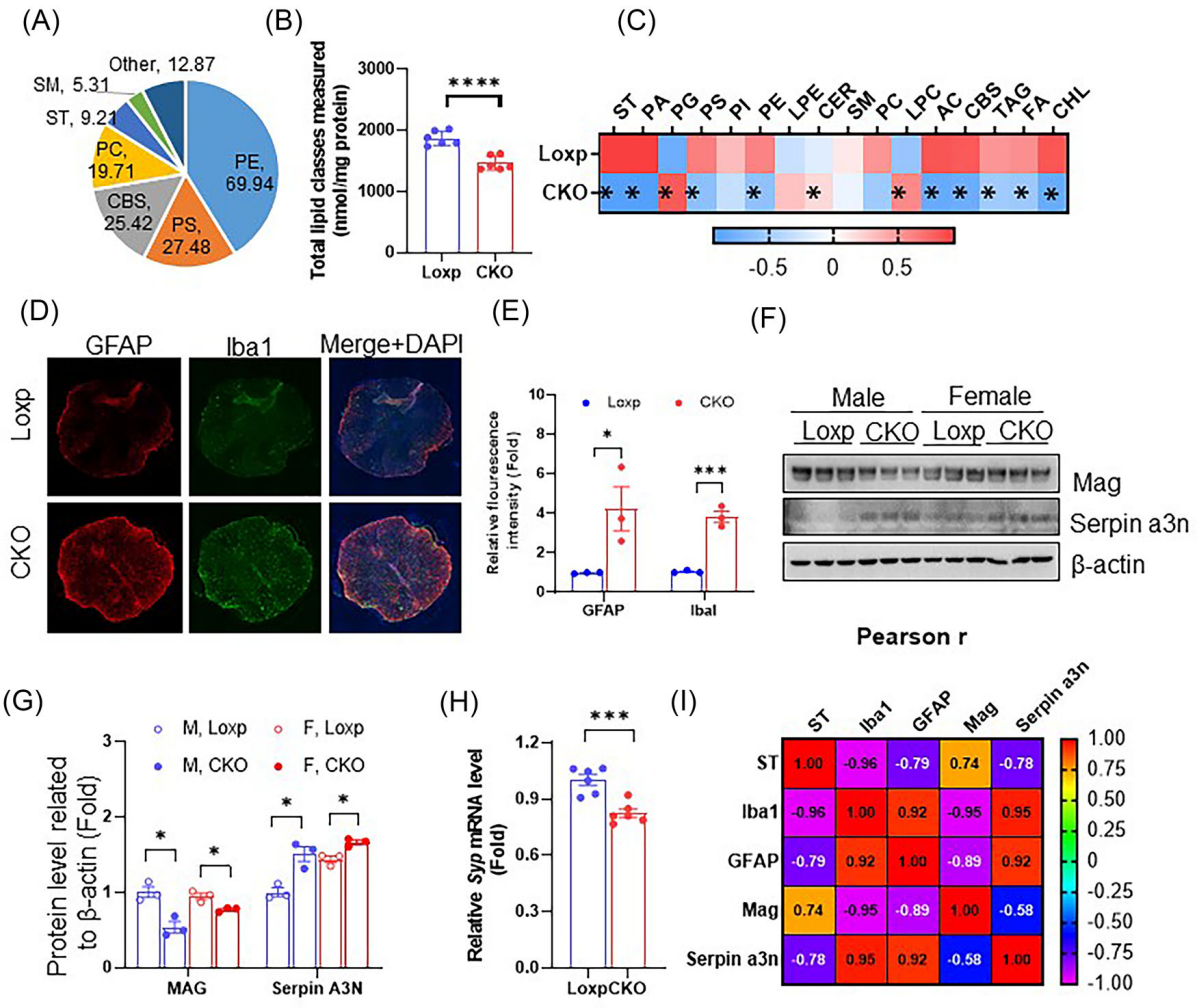
CNS sulfatide (ST) deficiency results in a lipidome and cell marker signature that resemble Alzheimer's disease condition in human. (A) Percentage content of major lipid species from total mouse spinal cord (for clear display, cholesterol is not included). (B) Total lipid content of spinal cord tissues from 20‐month‐old mice measured by shotgun lipidomics (*n* = 6 for each genotype). (C) Heatmap comparison between Loxp and CKO on levels of each lipid group from spinal cord samples. (D) Immunofluorescence staining of astrocyte marker glial fibrillary acidic protein, microglial marker Iba1 in spinal cord sections of Loxp and CKO mice at the age of 20 months (*n* = 3 animals for each genotype). (E) Quantification of (D) based on fluorescence intensity. (F) Western‐blot evaluation of oligodendrocyte marker Mag and astrocyte marker Serpina3n in mouse spinal cord tissues (*n* = 3/group). (G) Quantification of (F). (H) mRNA levels of synaptophysin in spinal cord samples of Loxp and CKO mice at the age of 20 months (*n* = 6/group) measured using NanoString. (I) Pearson correlation analysis of protein level association with ST levels in L_w tissue. *r* values are displayed for each association pair, all analysis have reached significance with *p* < .05. Data represent mean ± SEM, unpaired two‐tailed *t*‐test for (B), (E), (G) and (H), *p ≤ .05, ***p ≤ .001, ****p ≤ .0001.

Spinal cord is essential in modulating peripheral organ functions. Given the known function of ST in the brain and the severe loss of ST in AD spinal cord, we hypothesized that the spinal ST loss may contribute to peripheral organ dysregulation in AD. To investigate the consequences of ST loss on spinal cord integrity and function, we took advantage of an inducible *CST* conditional knockout mouse model.^18^ In mouse spinal cord tissue, we detected abundant CHL and PE followed by PS, CBS, PC, ST and SM (Figure 3A and Figure S2A). This is largely consistent with that of human spinal cord (Figure 1A and Figure S1B). Furthermore, ST deficiency resulted in significant decline on total spinal cord lipid content (Figure 3B), resembling the reduction of lipid content in AD (Figure 1B). Interestingly, the changes of lipid profiles induced by ST loss in mice (Figure 3C), including decrease of ST, PA, PS, PE, AC, CBS, TAG and FA, as well as increase of PG (Figure 3C and Figure S2B), were highly consistent to those in human AD spinal cord versus control (Figure 1F,G), suggesting that ST plays a critical role in maintaining spinal cord lipid homeostasis. This strongly indicated ST loss as the potential trigger for the lipidome changes in AD spinal cord. It is worth noting that no significant difference on total lipid content, or lipidome were detected in sciatic nerve between Loxp and CKO mice despite mild ST losses (Figure S2C,D), consistent with the lower expression of *Plp1* in the peripheral nervous system (PNS) versus CNS.

In human spinal cord, the coinciding observation of lipidome changes (Figure 1B,F,G) with protein marker signatures (Figure 1I) including synaptic dysfunction, microglial activation and myelin sheath disturbances, led us to hypothesize that lipid disruption may promote neuronal and glial consequences in AD. To test this, we measured cell markers in the spinal cord of ST‐deficient and control mice. Immunofluorescence staining showed marked enhancement of astrocyte marker glial fibrillary acidic protein (GFAP) and Iba1 signals in CKO mice (Figure 3D,E). In addition, reactive astrocyte marker serpin family A member 3N (SERPINA3N)^34^ was upregulated in CKO (Figure 3F,G), while myelin‐associated glycoprotein (MAG) (Figure 3F,G) and synaptophysin (Figure 3H) were decreased upon ST loss. Correlation analysis has further confirmed the strong association of these protein alterations with the levels of ST in AD spinal cord tissues (Figure 3I). Taken together, these results demonstrated that ST deficiency induces lipidome changes and neuroinflammation signature that closely resemble AD.

### 3.4 CNS ST deficiency results in severe neurological bladder disorder in mice

**FIGURE 4:**
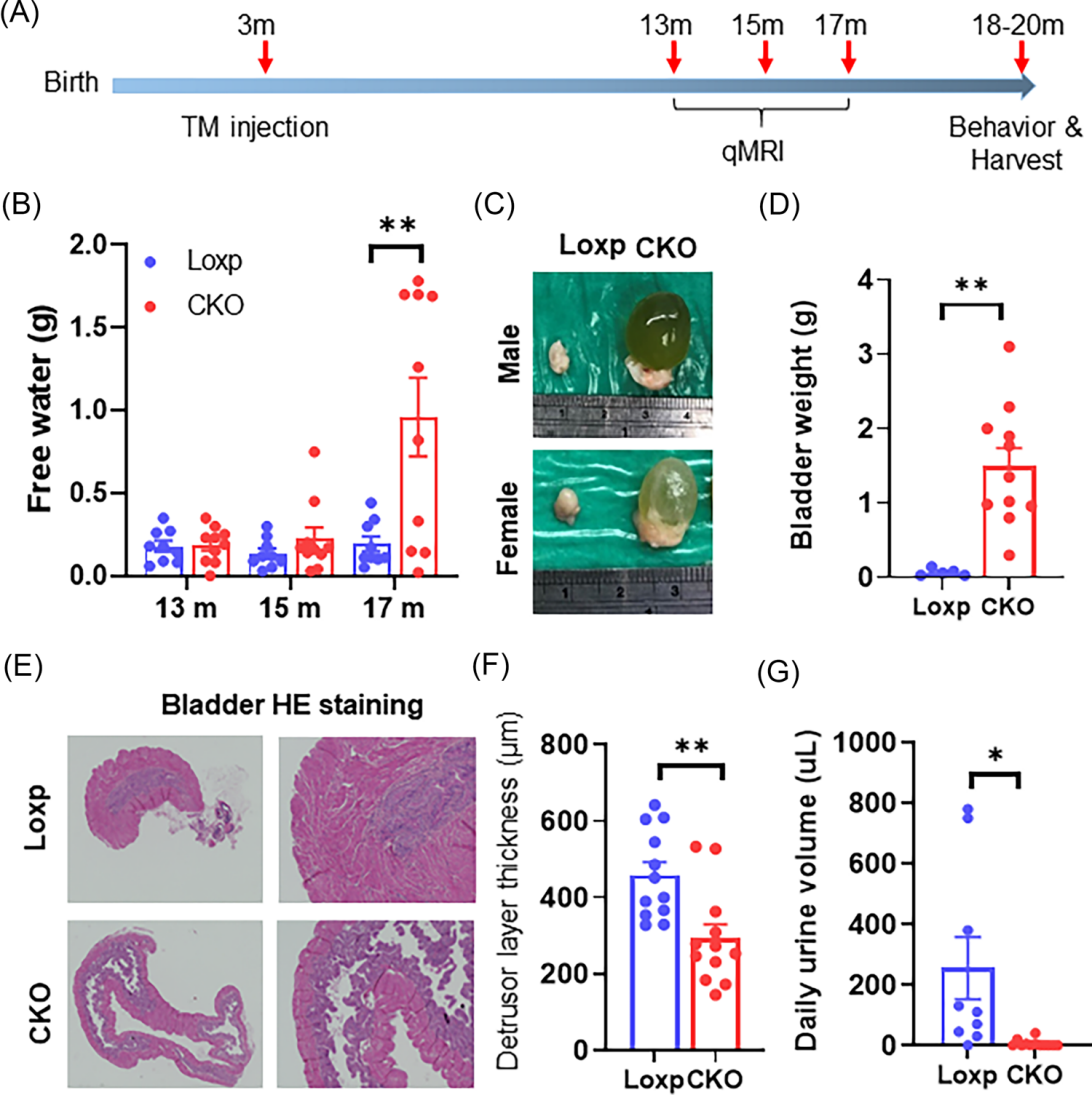
Adult‐onset myelin sulfatide (ST) deficiency leads to severe bladder enlargement. (A) Schematic timeline for the treatment and phenotyping of inducible myelin‐specific *CST* knockout mice. Loxp and CKO mice were injected with tamoxifen at 40 mg/kg dosage at the age of 3 mo, quantitative magnetic resonance imaging (qMRI) evaluation was performed at 10, 12 and 14 months post injection. Further behaviour test and tissue collection were done at 15−17 months post injection. (B) qMRI evaluation of free water content in mice at indicated age (*n* = 9 for Loxp, *n* = 10 for CKO). (C) Urinary bladder appearance, (D) bladder weight, (E) bladder tissue H&E staining and (F) detrusor layer thickness of bladder tissue from Loxp and CKO mice at the age of 20 months. (G) Daily urine volume measured using urine collection metabolic cage, mice were housed individually with free access to food and water during a 24‐h period (*n* = 9–12/group). Data represent mean ± SEM. Unpaired two‐tailed *t*‐test, *p ≤ .05, **p ≤ .01.

Our CNS ST deficiency mouse model closely resembled multiple aspects of human AD spinal cord (lipidome, gliosis and myelin destabilization), providing an ideal model to study peripheral effects of AD. We evaluated peripheral organ function in CKO mice at multiple time points post tamoxifen induction (Figure 4A). Surprisingly, qMRI showed a dramatic increase of free water content in CKO mice at the age of 17 months (Figure 4B). During tissue dissection, we found CKO mice had strikingly enlarged urinary bladders (Figure 4C), averaging ∼1.5 g heavier in urine weight compared to Loxp controls (Figure 4D), which consequently resulted in disruption of bladder morphology (Figure 4E) and a thinner detrusor layer (Figure 4F).

To further pinpoint the cause of bladder enlargement, we performed a metabolic cage study. Interestingly, CKO mice had greatly reduced daily urine volumes compared to Loxp littermates (Figure 4G) despite a comparable amount of water intake (Figure S3A). In addition, no significant change was detected in plasma creatinine levels (Figure S3B) or urine albumin levels upon knockout of *CST* (Figure S3C), suggesting CNS ST deficiency does not affect kidney function in terms of urine production. Moreover, frailty evaluation showed no significant differences between Loxp and CKO mice (Figure S3D). Grip strength, which reflects neuromuscular function, was similar between groups (Figure S3E). These observations demonstrated that bladder enlargement in ST deficient mice was not an outcome of overall health decline, kidney function change, or altered nerve–muscle communication. The root cause of this bladder phenotype appears to lie in the nervous system, a condition commonly known as neurogenic bladder.

The alternating phases of urinary bladder between continence and micturition are precisely controlled by spinal cord, brain and peripheral neural pathways.^35^ Although we aimed to deplete ST in the CNS using *Plp1*‐Cre, which mainly targets myelinating oligodendrocytes in the CNS, it is necessary to evaluate the potential impact on Schwann cells which express *Plp1* to a lower extent^36^ and form the myelin sheath in the PNS. To this end, we evaluated the function of peripheral nerves including tail, foot and sciatic nerves by electrophysiology. The electric signal travel latencies (Figure S4A–C) and amplitude (Figure S4D,E) of tail, foot (sural) and sciatic nerves were comparable between Loxp and CKO mice. Conduction velocity, a parameter reflecting speed of electrical signals propagate along nerves, also showed comparable levels between Loxp and CKO (Figure S4F–H). These results demonstrated that the loss of ST did not cause major changes in PNS function, suggesting the enlarged bladder in CKO mice was resulted from impaired CNS function.

### 3.5 CNS ST deficiency enhanced immune and inflammatory responses and suppressed neuronal and oligodendrocyte function

**FIGURE 5:**
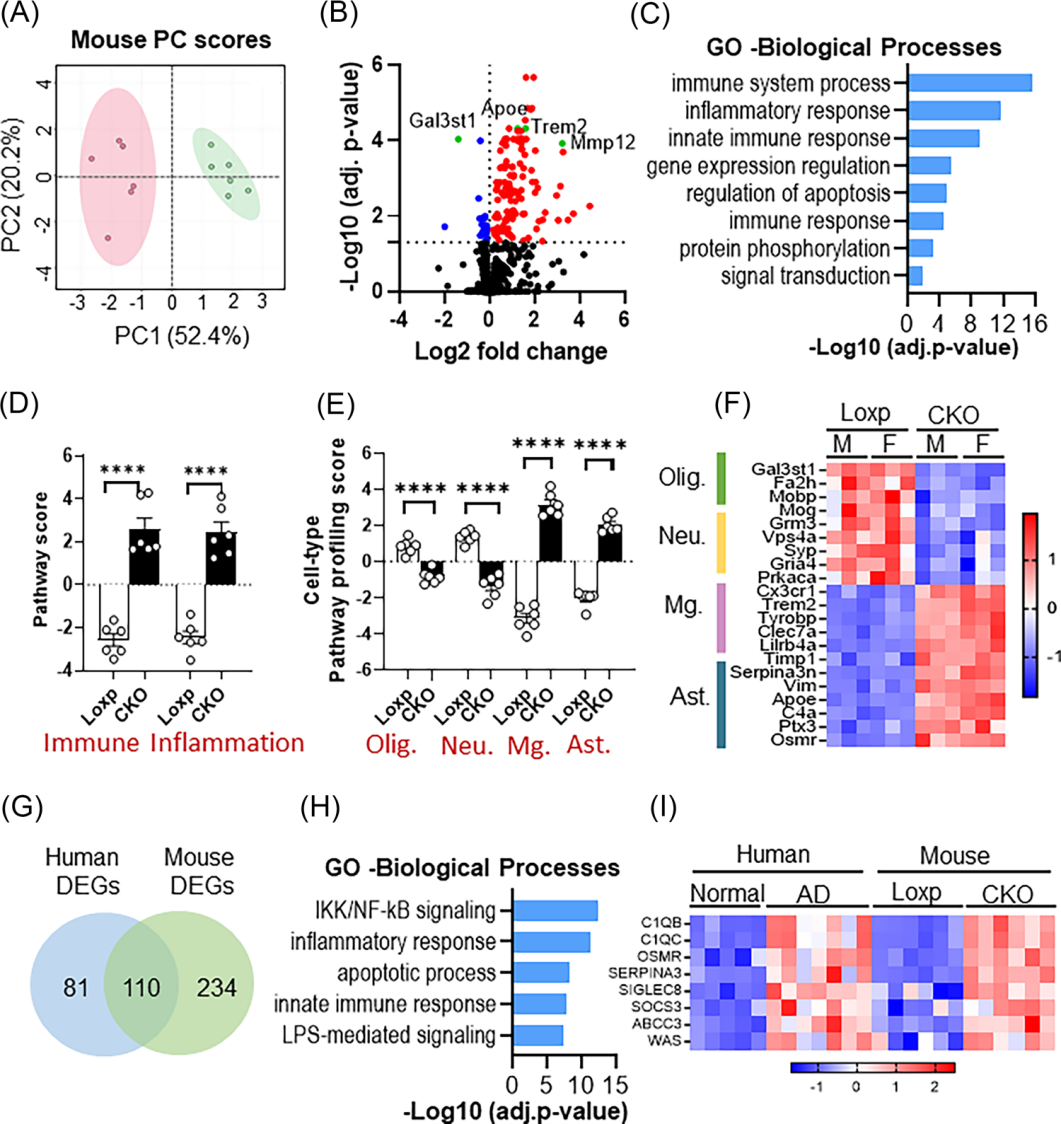
Sulfatide (ST) deficiency results in substantial changes on spinal cord gene expression profile. Spinal cord samples from 20‐month‐old Loxp and CKO mice were harvested for mRNA expression analysis using NanoString. (A) Principle component analysis (PCA) using all genes measured from NanoString (*n* = 3/genotype/sex, Loxp in green colour, CKO in pink colour). (B) Volcano plot displaying differentially expressed genes (DEGs) between Loxp and CKO mice. Red and blue coloured points represent induction and suppression in CKO versus Loxp, respectively, dotted line on *y*‐axis represent cut off FDR ≤ 0.05, top DEGs related to Alzheimer's disease (AD) were annotated indicated in green colour. (C) GO analysis showing top biological processes identified based on 143 significant DEGs between Loxp and CKO. (D) NanoString pathway score analysis for inflammatory and innate immune response and (E) cell type‐specific function. (F) Heatmap showing DEGs between Loxp and CKO mice from NanoString grouped based on cell type related functions (*n* = 3/genotype/sex). (G) Venn diagram showing overlap DEGs between human (AD vs. normal) and mouse (CKO vs. Loxp) Nanostring using *p*‐value ≤ .05. (H) Human lumbar spinal cord (mixture of white and grey matter) from normal (*n* = 5) and AD (*n* = 7) individuals were tested using Nanostring neuroinflammation panel. GO analysis showing top biological processes identified based on 191 DEGs (using *p*‐value ≤ .05). (I) Heatmap comparison of mRNA levels on the eight shared top DEGs between human and mouse NanoString results. Data represent mean ± SEM, unpaired two‐tailed *t*‐test, ****p ≤ .0001.

To unravel the consequences of CNS ST loss at a molecular level, we assessed the gene expression profile of Loxp and CKO mice spinal cord tissue using a NanoString neuroinflammation panel which consists of 770 unique gene targets. PCA analysis showed clear separation of Loxp from the CKO group (Figure 5A). One hundred and forty‐three differentially expressed genes (DEGs) were detected between the Loxp and CKO groups (fold change ≥1.2; *p‐adj*. ≤ .05), among which 118 were upregulated and 25 were downregulated (Figure 5B). Inquiring the most significantly DEGs (top 30 with lowest *p‐adj*.) against GWAS Catalog 2019 database revealed high relevance to AD (data not shown). Specifically, AD risk genes *Apoe*, *Trem2* and *Mmp12* were greatly induced in CKO spinal cord (Figure 5B). Gene ontology (GO) analysis with all 143 DEGs showed inflammatory and immune responses among top altered biological processes in the CKO mice (Figure 5C). A UniprotKB keywords analysis identified these genes mostly encoded for membrane and secreted factors which function as receptors and cytokines (Figure S5A,B), and were involved in phagocytosis, chemokine and interleukin pathways according to a WikiPathways query (Figure S5C). Consistently, pathway score analysis using nSolver software showed great induction of adaptive immune response and inflammatory signalling in CKO versus Loxp (Figure 5D). Additionally, cell type‐specific pathway profiling detected significant increases of microglial and astrocytic function, contrasted by decreases of oligodendrocyte and neuron‐related pathways in the CKO samples (Figure 5E). The changes in cellular function that resulted from ST loss were further demonstrated by specific cell markers levels shown in Figure 5F, from which similar conclusions can be drawn for both sexes. We observed increased microglial activation (e.g., *Cx3cr1, Trem2* and *Tyrobp*), enhanced astrogliosis (e.g., *Serpina3n, Apoe, Vim* and *Osmr*), and decreased oligodendrocyte markers (e.g., *Mobp* and *Mog*), indicating myelin disruption, and decline of neuron function‐related markers (e.g., *Syp, Gria4* and *Prkaca*).

To comprehensively compare effects of ST loss between mouse and human, we further measured the gene expression profile in lumbar spinal cord of AD patients and normal individuals using NanoString human neuroinflammation panel. A total of 191 genes were changed (fold change ≥1.2) between normal and AD with *p‐value* less than .05, within which 110 were overlapped with mouse gene DEGs (CKO vs. Loxp) (Figure 5G). GO analysis and UniprotKB analysis identified most of these genes encode for membrane proteins involved in protein binding during the biological processes of inflammatory regulation and immune response (Figure 5H and Figure S5D,E).

Among the top 15 DEGs of human AD versus normal (*adj. p* ≤ .05), 8 were overlapped with mouse DEGs (CKO vs. Loxp, *adj. p* ≤ .05) (Figure S5F), including microglial markers *C1QC* and *C1QB*; astrocyte marker *OSMR*, *SERPINA3*; transporter gene *ABCC3*; inflammation‐regulatory gene *SIGLEC8* and *SOCS3* ^37^; and a cytoskeleton modulator gene *WAS* ^38^ which suggest aberrant sprouting of neurites^39^ and alteration in microglial phagocytosis function^40^ (Figure 5I). Taken together, the shared transcriptomic signature between human and mouse demonstrated consistency on the induction of immune and inflammatory responses and the decline of neuron and myelin functions, providing evidence on the causal role of ST deficiency on AD‐related transcriptome alterations.

### 3.6 ST deficiency promotes AD‐like lipidome alterations via disruption of lipid metabolism‐related genes

**FIGURE 6:**
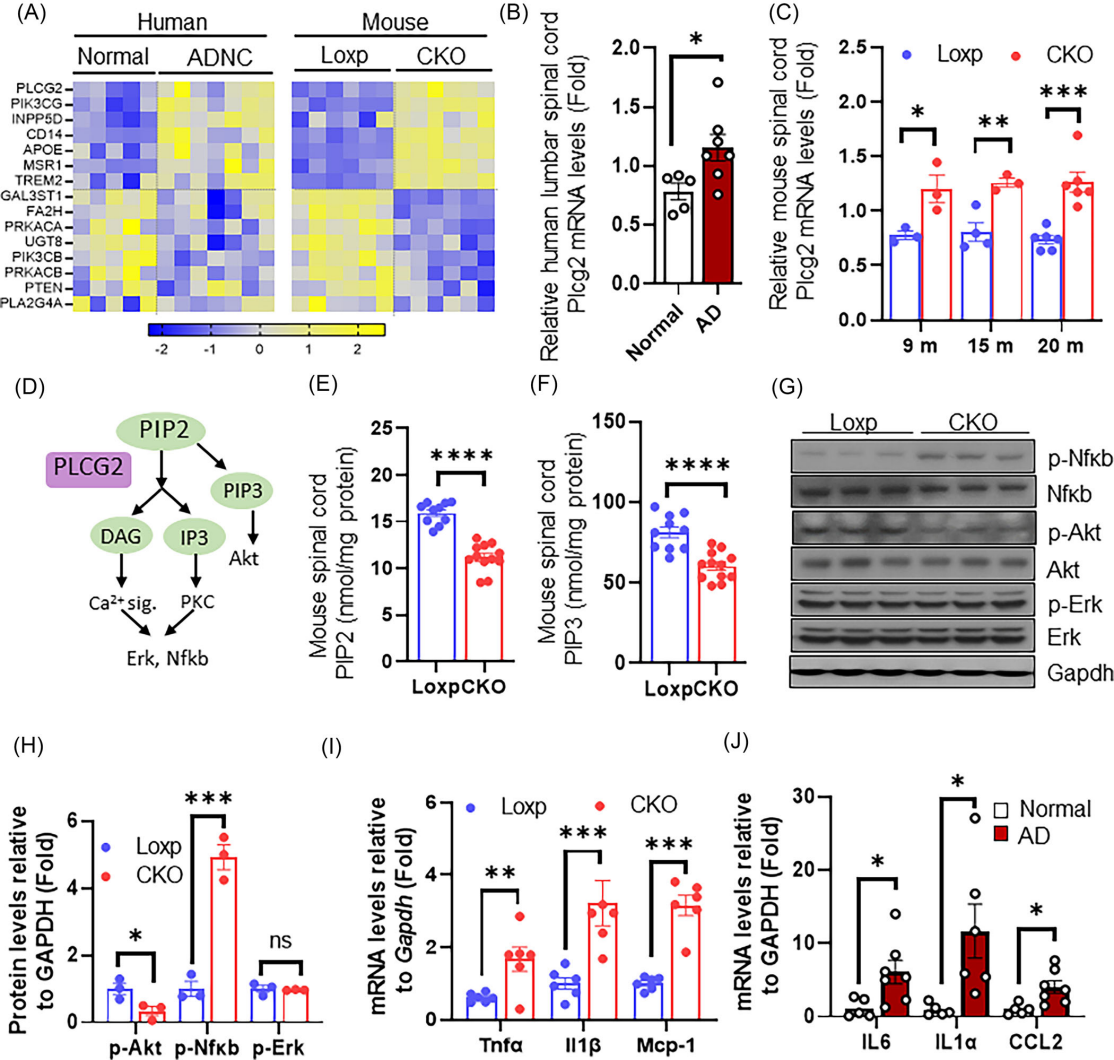
Sulfatide (ST) loss disrupts spinal cord lipid metabolism and associated inflammatory signaling pathways. (A) Lipid metabolism gene mRNA levels in human lumbar spinal cord (*n* = 5 for normal, *n* = 7 for Alzheimer's disease [AD]) and mice spinal cord (*n* = 6/group) detected using NanoString. (B) Comparison of phospholipase C γ2 (PLCG2) expression levels between normal and AD human lumbar spinal cords based on mRNA counts (*n* = 5 for normal, *n* = 7 for AD). (C) mRNA levels of PLCG2 in spinal cord samples of Loxp and CKO mice at the ages of 9, 15 and 20 months (*n* = 3–6/group) measured using NanoString. (D) Brief diaphragm of PLCG2‐associated signaling. (E) PIP2 and (F) PIP3 levels of mouse spinal cord tissues at 20 months old evaluated by shotgun lipidomics. (G) Western blot of PLCG2‐associated cellular signaling in 20‐month‐old mouse spinal cord samples. (H) Quantification of (G). qPCR evaluation of NFκB target genes in (I) mouse and (J) human spinal cord. Data represent mean ± SEM. Unpaired two‐tailed *t*‐test, *p ≤ .05, **p ≤ .01, ***p ≤ .001, ****p ≤ .0001.

Finally, we sought to understand the mechanism on how ST loss can cause wide‐spread lipidome changes and inflammation in the spinal cord. To this end, we detected significant alterations of multiple lipid metabolism regulators/enzymes such as phospholipase C γ2 (*PLCG2*), *INPP5D, PIK3CG, FA2H, UGT8* and *PLA2G4A* as well as lipid binding/transporting proteins including *APOE, CD14, MSR1* and *TREM2* (Figure 6A). An inquiry into published human spinal cord single cell sequencing data^41^ showed that these lipid metabolism genes are mainly expressed in populations of oligodendrocytes and microglial cells (Figure S6A–I). Among these genes, fatty acid 2‐hydroxylase (Fa2h) is specifically expressed by oligodendrocytes and functions to synthesize 2‐hydroxylated fatty acid that are important for the formation of normal myelin.^42^, ^43^ In addition, some genes from this list are involved in both lipid metabolism as well as inflammation pathways. For example, we found marked and early induction of microglial AD risk gene *PLCG2* ^44^ in the spinal cord of human (AD vs. normal) and mouse (CKO vs. Loxp) (Figure 6B,C). *PLCG2* encodes an intracellular enzyme that cleaves the membrane phospholipid phosphatidylinositol‐4,5‐bisphosphate (PIP2) to diacylglycerol (DAG) and inositol‐1,4,5‐trisphosphate (IP3) upon activation of various transmembrane immune receptors (e.g., TREM2).^45^ The resulting products IP3 and DAG regulate survival, phagocytosis and cytokine production via NFκB and MAPK/ERK activation. Alternatively, PIP2 converts into PIP3 to promote growth and autophagy via AKT signaling^45^ (Figure 6D). Interestingly, while *PLCG2* expression was induced, shotgun lipidomics detected significant reduction of both PIP3 and PIP2 in spinal cord of *CST* knockout mice (Figure 6E,F). In the meantime, we found ST loss enhanced NFκB phosphorylation and suppressed AKT phosphorylation (Figure 6G,H). Consistently, we detected induction of NFκB downstream inflammatory cytokines in both human (AD vs. normal) and mouse (CKO vs. Loxp) spinal cord tissues, including *IL6, IL1α, IL1β, TNFα* and *MCP‐1* (Figure 6I,J). These observations suggest that ST deficiency greatly upregulated PLCG2‐associated lipid metabolism cascade as well as downstream NFκB inflammatory pathway, both of which may further promote tissue‐wide lipidome disruption and inflammatory response involving multiple cell types.

## 4 DISCUSSION

The involvement of spinal cord in AD has only attracted attention recently, yet most of these studies have focused on evaluating traditional AD markers discovered from AD brain studies.^9^, ^11^ Essential factors that trigger spinal cord function change and its causal relationship to peripheral tissue dysregulation in AD have yet to be fully understood.

Here we employed quantitative shotgun lipidomics and identified a unique lipid signature in post‐mortem AD spinal cord characterized by marked reduction of myelin lipids. We further identified myelin‐enriched ST among the most reduced lipids in AD. Using a mouse model of adult‐onset myelin ST deficiency, we demonstrated that ST deficiency is sufficient to trigger a lipid profile similar to that of AD. Moreover, loss of CNS ST impaired spinal cord function and consequently led to severe bladder disorder, a common peripheral symptom among late‐stage AD patients. Mechanistically, we found ST loss promotes a transcriptional program that induces immune/inflammation response and substantially disrupts lipid metabolism in human and mouse spinal cord tissue.

Dysregulation of lipid metabolism is an important risk factor for the initiation and progression of AD.^46^ Recent GWAS studies have identified multiple AD risk loci which involve in lipid processing (such as *APOE, TREM2, PLCG2, ABCA1, ABCA7* and *INPP5D*).^47^, ^48^ Furthermore, a growing body of literature has associated brain lipid disruption with AD pathology.^20^, ^28^ Seeking to understand the lipid dynamics in spinal cord tissue in AD, the current study found substantial reduction of overall lipid content as well as decline of multiple lipid species in AD versus normal. Among the altered lipid classes, important membrane‐forming glycerophospholipids such as PE and PS were decreased in both AD and ST‐deficient mouse spinal cord. This is consistent with studies showing decreased phospholipid contents in AD post‐mortem brains.^49^, ^50^ As the most abundant membrane lipids, phospholipid losses may contribute to impaired CNS function through reduced neuronal membrane fluidity, restricted membrane protein diffusion, altering protein–protein interaction and changes in neuronal signaling.^51^ We also observed a decreased FA and unsaturation index in AD and CKO mice. This is in line with lower unsaturated FA in AD versus control brains^52^ and in aging.^53^ These changes may promote age‐ and disease‐related neuronal deterioration through mitochondrial dysfunction, oxidative stress and altering neuronal membranes.^54^ Notably, we found a decline of CBS and CHL species in AD spinal cord, both of which have been shown to be important for myelin function.^55^, ^56^ Interestingly, these lipid species were also downregulated by ST deletion in the CKO mouse model. Based on these observations, we believe ST loss may function as a triggering factor to induce lipidome alterations, including the decline of CBS and CHL, which collaboratively contribute to the spinal cord myelin destabilization in late‐stage AD.

An important note on our lipidome study was the identification of ST reduction in human AD spinal cord versus the normal group. We chose to focus our study on ST based on the following lines of evidence: (1) change of AD spinal cord lipids happened mainly in myelin, where ST is a major class of lipids; and (2) strong involvement of ST in AD brain demonstrated by our previous work^18^, ^20^ suggesting ST may also play a role in other components of CNS, including spinal cord. ST constitutes 2%−10% of the myelin lipid pool and are synthesized by oligodendrocytes by sulfating CBSs via *CST* (encoded by *Gal3st1* gene). We previously identified ST depletion in AD brain at early disease stages, while other major classes of lipids (such as PC, PE, PS) remained intact.^20^ Here our observation of ST depletion in AD spinal cord further affirmed this association and illustrated that phospholipids, including PE and PS, were disrupted as a consequence of long‐term ST deficiency. A query of human AD versus control post‐mortem brain single cell study revealed altered expression of many genes involved in myelin lipid synthesis and metabolism in OLGs of AD subjects^55^, ^57^ (e.g., upregulation of *Plpp2* presumably leading to reduced levels of phosphatidic acid, a key substrate for all phospholipid biosynthesis, and downregulation of *ABCA2*, which is known to affect sphingolipid metabolism^58^). These changes are well in line with the altered lipid metabolism pathway induced by ST deficiency in mice.

The lipid profile induced by ST loss in our mouse model was nearly identical to that of human AD, strongly indicating that ST depletion induced myelin destabilization and wide‐spread lipidome disruption may be a critical contributor to AD‐related peripheral organ diseases. This is also evident by decreased myelin protein markers (MOBP and MAG), and in line with other studies suggesting AD as a disease with myelin deterioration.^59^, ^60^ The finding of ST loss as a trigger for such changes provides a mechanism for better understanding AD initiation.

Our study demonstrated that CNS ST loss was sufficient to cause neurogenic bladder. While the voluntary micturition cycle involves the brain (cerebral cortex and pons) and spinal cord (mainly lumbar and sacral cord), it is challenging to pinpoint the exact contribution of each component. Although our CNS ST‐deficient mouse model cannot differentiate the contribution by brain versus spinal cord, here we provided strong evidence emphasizing the critical involvement of spinal cord dysfunction in AD‐associated bladder disorders. Numerous studies have demonstrated the consequence of spinal cord disease^61^, ^62^, ^63^ which leads to neurogenic bladder through myelin^63^ or nerve damage.^61^, ^62^ Interestingly, both myelin disruption and neuronal deterioration were prominent in spinal cord tissue of ST‐deficient mouse model, strongly supporting the notion that ST loss‐induced spinal cord alterations contribute to neurogenic bladder in humans and mice.

Following ST loss, we observed lipidome‐wide changes (mostly contributed by myelin) as well as activation of immune and inflammation responses. However, the portion contributed by either event to downstream effects (e.g., bladder dysregulation) is still unclear. Our previous study demonstrated that in the early ages (4.5 and 9 months post tamoxifen injection) of the CNS ST loss model, no significant impact was detected on myelin homeostasis, despite the observation of clear microglial and astrocytic activation^18^ at these ages. On the other hand, our current study showed that prolonged CNS ST loss (17 mo post tamoxifen injection) led to myelin perturbation accompanied by lipidome alterations, which is in concordance with the rise of the neurogenic bladder phenotype. This may suggest that initial activation of immune and inflammation responses were not sufficient to cause loss of bladder control, while late‐stage myelin lipidome disruption seems to be the essential turning point that causes spinal cord dysfunction. Nevertheless, changes of lipid metabolism and neuroinflammation responses are known to be closely correlated and facilitate one another. For example, microglia‐enriched TREM2 was induced upon ST loss. As TREM2 is a membrane receptor that can sense lipids (including ST) as its ligand,^64^ decline of ST level may induce compensatory induction of TREM2 receptor expression. As TREM2 is known to be critical for final‐stage disease‐associated microglia (DAM) transition,^65^ this would eventually lead to enhanced microglial activation and promote neuroinflammation. Conversely, it is also well established that inflammatory factors can modulate lipid metabolism in multiple aspects,^66^ including uptake, consumption and lipid enzyme function. Particularly, some factors are involved in both lipid metabolism and inflammation processes. In support of this, we identified *PLCG2* among the first early responding factors to ST deletion. As a microglia‐enriched AD risk gene^44^, ^48^ and a lipid metabolism modulator, *PLCG2* is induced in AD, associated with inflammation^67^ and its polymorphism has been found to affect AD incidence.^68^ Functioning closely with TREM2, the TREM2‐PLCG2 cascade transforms extracellular stimuli into microglial intracellular signalling to promote transcription of inflammatory factors.^69^ Consistently, in our CNS ST loss mouse model, PLCG2 lipid substrate PIP2 was decreased while multiple PLCG2‐NFκB downstream genes were induced. Future studies using TREM2 or PLCG2 inhibition/deletion models are necessary to pinpoint the exact involvement of these factors in AD‐related peripheral disease progression.

Using a *CST* deletion mouse model, we demonstrated that targeting ST synthesis enzyme can effectively downregulate ST levels. This suggests that it is possible to develop enzyme‐targeting strategies to manipulate ST levels for disease treatment purpose. Indeed, we believe a CST or ceramide galactosyltransferase (CGT, mediates enzymatic step before CST for ST synthesis) activator may be effective to bring up ST levels back to normal. In addition, inhibition of arylsulfatase A (ARSA), the enzyme that mediates the degradation of ST, may also help to counteract ST loss. We believe our study provides a basis for future study on developing strategies to achieve ST homeostasis to prevent disease development.

## 5 CONCLUSIONS

**FIGURE 7:**
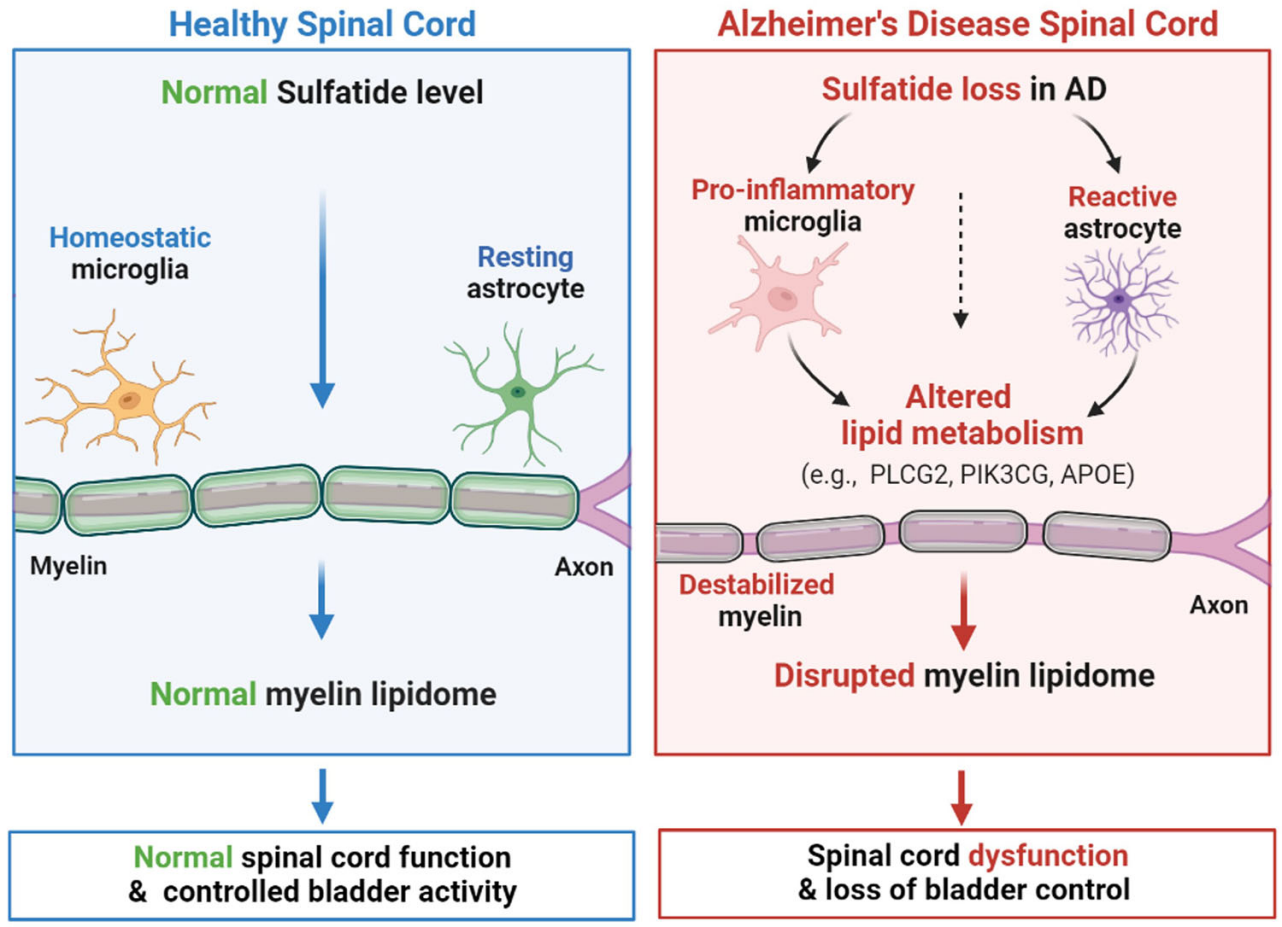
Graphic summary of the role of sulfatide (ST) in regulating spinal cord function in Alzheimer's disease (AD). Under normal condition, sufficient ST levels are critical for maintaining a stable myelin lipidome in order to facilitate physiological spinal cord activity in controlling peripheral organ functions. Under disease condition such as early‐stage AD, substantial loss of ST promotes neuroinflammation and induces altered lipid metabolism. These changes consequently result in disrupted myelin lipidome, which contributes to dysregulated spinal cord function along with loss of bladder control in middle–late stages of AD (graph created using BioRender).

In summary, our study has for the first time identified a marked reduction of spinal cord lipids in AD versus normal individuals. Using an adult‐onset myelin ST deficiency mouse model, we further elucidated ST deficiency as a triggering factor for neuroinflammation and lipidome disruption, which was sufficient to cause spinal cord dysfunction and bladder dysregulation (Figure 7). Our findings provide a novel prospective regarding AD‐associated peripheral organ dysregulation and indicate monitoring or modulating ST levels may be a promising approach for detecting and combating AD‐related peripheral diseases.

## 6 LIMITATIONS

The current study had limited number of cases for human spinal cord sample studies due to sample availability, we were not able to perform a sex‐specific analysis due to this limitation. In addition, the incidence of bladder dysfunction among human AD patients can be variable, unfortunately, patient bladder function was not documented upon post‐mortem spinal cord sample collection. Lastly, by utilizing a CNS ST loss mouse model in this study, we did not exclude the possible participation of brain ST loss in promoting bladder dysfunction.

## CONFLICT OF INTEREST STATEMENT

The authors declare no conflicts of interest.

## Supporting information

Supporting Information
