# Supplementary material for: Central nervous system sulfatide deficiency as a causal factor for bladder disorder in Alzheimer's disease
Source: Clin Transl Med. 2023 Jul 21;13(7):e1332. doi: 10.1002/ctm2.1332 (PMC10361545; doi:10.1002/ctm2.1332)
Supplement: Supplementary file 1 — Supporting Information [file CTM2-13-e1332-s001.docx]

**Supporting Information for**

**Central nervous system sulfatide deficiency as a causal factor for bladder disorder in Alzheimer’s disease**

Sijia He^1^, Shulan Qiu^1^, Meixia Pan^1^, Juan P Palavicini^1,2^, Hu Wang^1^, Xin Li^1^, Anindita Bhattacharjee^1^, Savannah Barannikov^3^, Kevin F Bieniek^3^, Jeffrey L Dupree^4,5^, and Xianlin Han^1,2,*^

***Corresponding author:** Xianlin Han. Email: [hanx@uthscsa.edu](mailto:hanx@uthscsa.edu)

**This file includes:**

Supporting text

Figures S1 to S6

Tables S1 to S2


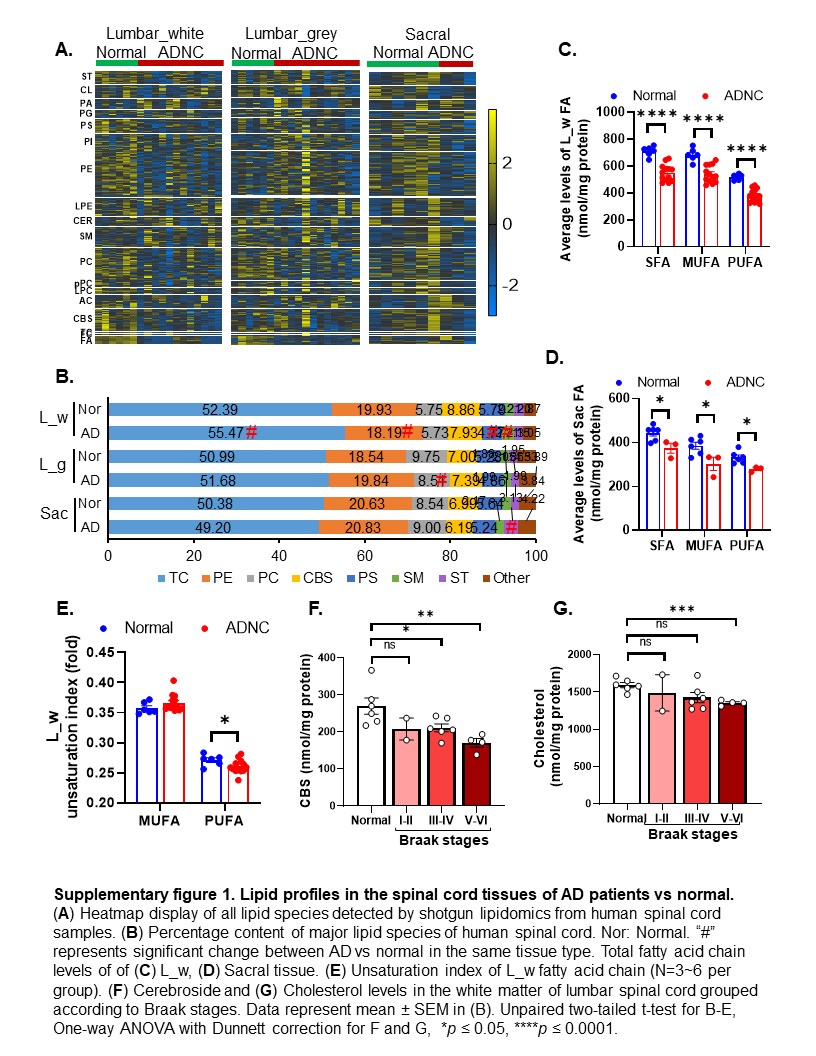


**Fig. S1. Lipid profiles in the spinal cord tissues of AD patients vs normal subjects.**

(A) Heatmap display of all lipid species detected by shotgun lipidomics from human spinal cord samples. (B) Percentage content of major lipid species of human spinal cord. Nor: Normal. “#” represents significant change between AD vs normal in the same tissue type. Total fatty acid chain levels of of (C) L_w, (D) Sacral tissue. (E) Unsaturation index of L_w fatty acid chain (N=3~6 per group). (F) Cerebroside and (G) Cholesterol levels in the white matter of lumbar spinal cord grouped according to Braak stages. Data represent mean ± SEM in (B). Unpaired two-tailed t-test for B-E, One-way ANOVA with Dunnett correction for F and G, *p ≤ 0.05, ****p ≤ 0.0001.
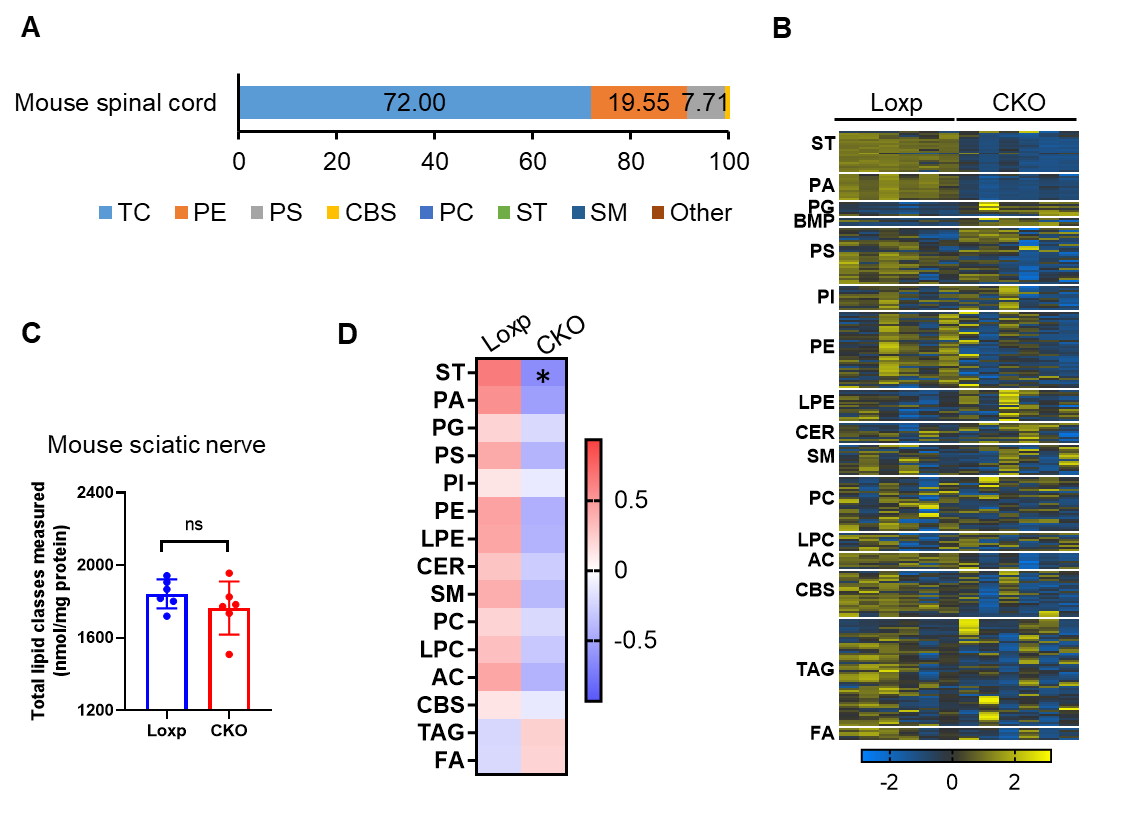


**Fig. S2. Levels of lipids in spinal cord and sciatic nerve tissues of Loxp and CKO mice.**

(**A**) Percentage content of major lipid species from total mouse spinal cord. (**B**) Heatmap display of all lipid species detected by shotgun lipidomics from mouse spinal cord (20m old, n = 3/sex/genotype). (**C**) Total lipid content of sciatic nerve tissue quantified in relative to protein levels (20m old, n=6 for each group). (**D**) Heatmap comparison between loxp and CKO on levels of each lipid group from sciatic nerve samples (20m old, n=6 for each group). Data represent mean ± SEM, Unpaired two-tailed t-test, *p ≤ 0.05, n.s. mean not significant.


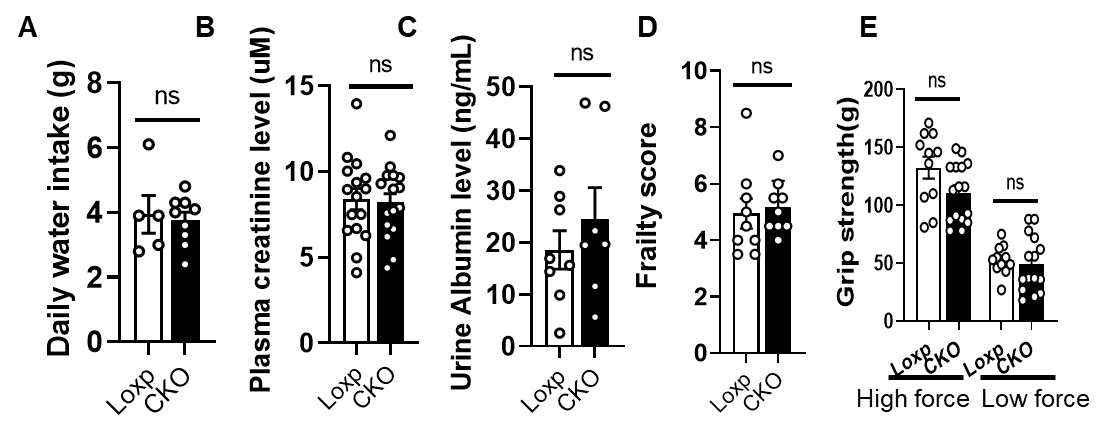


**Fig. S3. Evaluation of the impact of sulfatide loss on renal function, and neuromuscular function in mice.** (**A**) Daily water intake of Loxp and CKO mice at 18 m measured by metabolic cage. (**B**) Plasma Creatinine level, and (**C**) Urine albumin level of control and ST deficient mice at 20 m measured using Elisa. (**D**) Frailty score and (**E**) Grip strength test of Loxp and CKO mice at age of 18 m (n = 9~16/group). Data represent mean ± SEM, Unpaired two-tailed t-test, n.s. mean not significant.


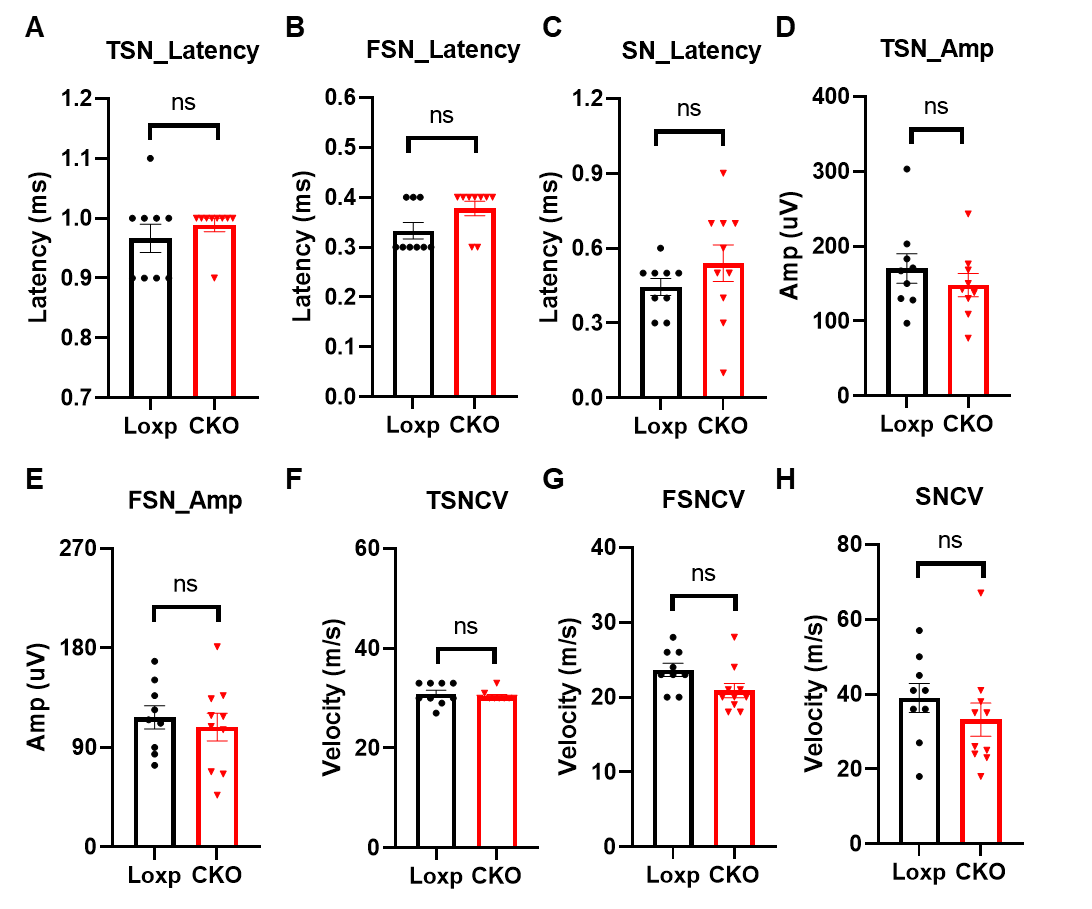


**Fig. S4. Knockout of sulfatide does not affect PNS function.** Loxp and CKO mice of 18 m age were used for measure peripheral nerve function. Latency of (**A**) tail sensory nerve (TSN), (**B**) foot sensory nerve (FSN), and (**C**) sciatic nerve (SN). Amplitude of (**D**) tail sensory nerve, and (**E**) foot nerve. Conduction velocity of (**F**) tail sensory nerve, (**G**) foot nerve, and (**H**) sciatic nerve. n = 9 for Loxp, n = 10 for CKO. CV: Conduction velocity. Data represent mean ± SEM, Unpaired two-tailed t-test, n.s. mean not significant.


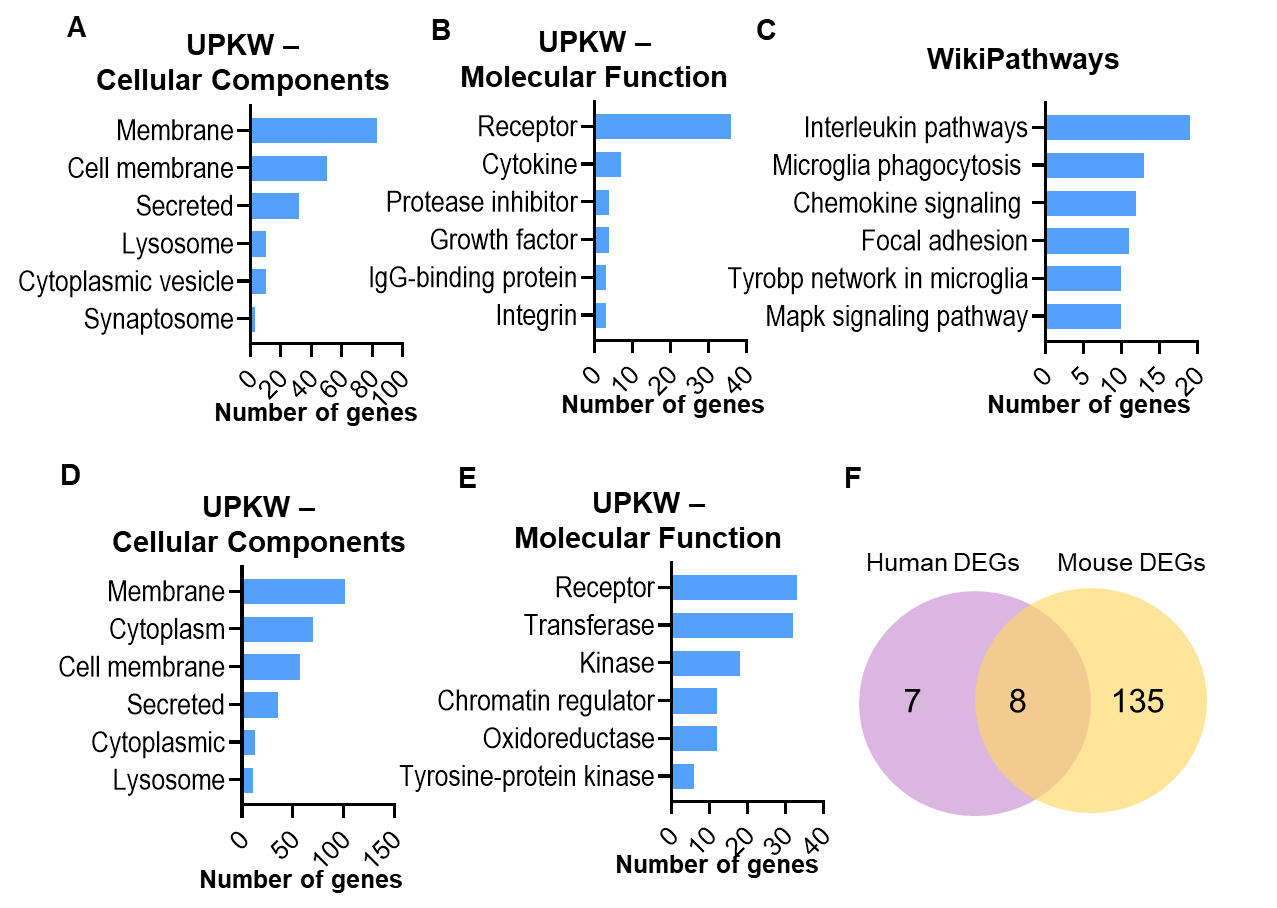


**Fig. S5. Database analysis of mouse and human Nanostring.** (**A**) UniprotKB keywords analysis on cellular components, (**B**) UniprotKB keywords analysis on molecular function, and (**C**) WikiPathway analysis for the143 significant DEGs detected between Loxp and CKO mouse spinal cord tissue. (**D**) UniprotKB keywords analysis on cellular components. (**E**) UniprotKB keywords analysis on molecular function for the191 significant DEGs detected between AD vs Normal human spinal cord tissues. (**F**) Venn diagram showing overlap DEGs between human (AD vs Normal) and mouse (CKO vs Loxp) Nanostring using adj. p-value ≤ 0.05.


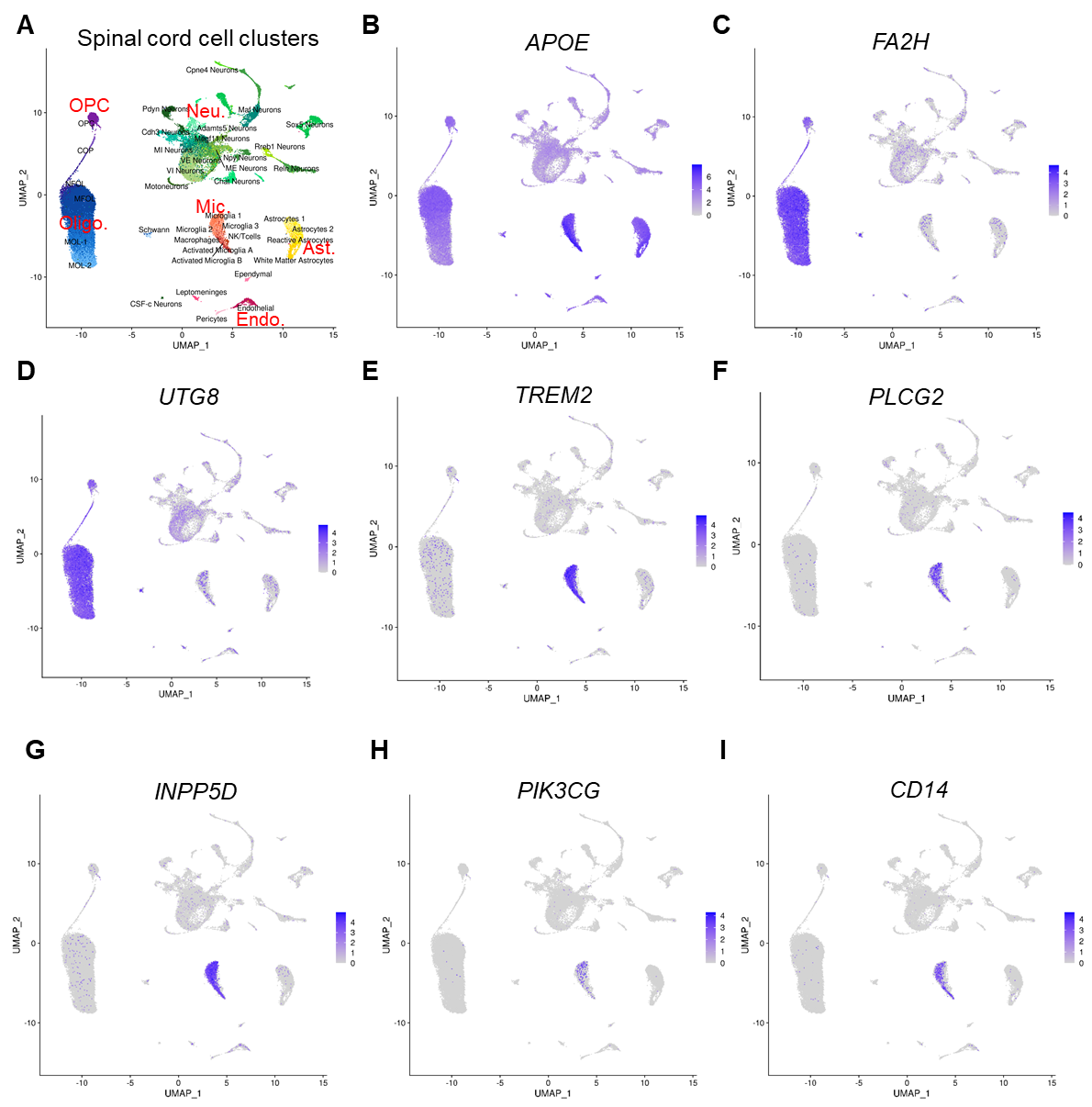


**Fig. S6. mRNA distribution of lipid metabolism genes in different cell populations from human spinal cord tissue.** Single cell sequencing database inquiry was performed using resource from (https://seqseek.ninds.nih.gov/spinalcordinjury) (DIO: 10.1038/s41467-021-25125-1). (**A**) UMAP display of cell populations detected in human spinal cord tissue by singel cell sequencing. mRNA levels of (**B**) *APOE*, (**C**) *FA2H*, (**D**) *UGT8*, (**E**) *TREM2*, (**F**) *PLCG2*, (**G**) *INPP5D*, (**H**) *PIK3CG*, and (**I**) *CD14*.

**Table S1. List of primers used**

| **Primer name** | **5'-sequence-3'** |
| --- | --- |
| **m-GAPDH-F** | GAGAAACCTGCCAAGTATG |
| **m-GAPDH-R** | GGAGTTGCTGTTGAAGTC |
| **m-IL1b-F** | GAAATGCCACCTTTTGACAGTG |
| **m-IL1b-R** | TGGATGCTCTCATCAGGACAG |
| **m-MCP1-F** | TGATCCCAATGAGTAGGCTGGAG |
| **m-MCP1-R** | ATGTCTGGACCCATTCCTTCTTG |
| **m-TNFα-F** | GCCTCTTCTCATTCCTGCTT |
| **m-TNFα-R** | CTCCTCCACTTGGTGGTTTG |
| **h-GAPDH-F** | CATGTTCCAATATGATTCCACC |
| **h-GAPDH-R** | CTCCATGGTGGTGAAGACGC |
| **h-IL6-F** | CCTAGAGTACCTCCAGAACAG |
| **h-IL6-R** | CTTCGTCAGCAGGCTGGCA |
| **h-IL1a-F** | ATCAGTACCTCACGGCTGCT |
| **h-IL1a-R** | TGGGTATCTCAGGCATCTCC |
| **h-CCL2-F** | GCTCAGCCAGATGCAATCAA |
| **h-CCL2-R** | TTCTTTGGGACACTTGCTGC |

**Table S2. List of antibodies used**

| **Antibody** | **Source** | **Identifier** |
| --- | --- | --- |
| **MOBP** | Proteintech | 12472-1-AP |
| **Synaptophysin** | CST | 36406T |
| **Iba1/AIF1(western-blot)** | FUJIFILM Wako | 016-20001 |
| **GAPDH** | CST | 2118L |
| **GFAP** | Millipore | AB5541 |
| **Iba1(immunoflourescence)** | FUJIFILM Wako | 019-19741 |
| **Mag** | CST | 9043S |
| **Serpin a3n** | R&D systems | AF4709 |
| **β-actin** | CST | 4970S |
| **p-Akt** | CST | 9271T |
| **p-Erk** | CST | 4370S |
| **p-Nfκb** | CST | 3033T |
